# Estrous cycle modulates fasting-induced torpor propensity via hypothalamic estrogen signalling

**DOI:** 10.1038/s41598-026-41051-y

**Published:** 2026-02-27

**Authors:** Christopher J. Marshall, Anthony E. Pickering, Michael T. Ambler

**Affiliations:** Anaesthesia, Pain and Critical Care Research, School of Physiology, Pharmacology & Neuroscience, University of Bristol, Bristol, BS8 1TD UK

**Keywords:** Neuroscience, Physiology, Zoology

## Abstract

Torpor is a state of transient hypometabolism and hypothermia that is engaged by many species in adverse conditions such as food scarcity. Chemo- or optoactivation of neurons in the hypothalamic preoptic area (POA) drives torpor-like hypometabolism and hypothermia. Estrogens, principally estradiol, modulate thermogenesis and energy balance by central actions, and POA neurons express the canonical estrogen receptor ERα. We explored the role of POA estrogen signalling in fasting-induced torpor in the mouse. We found that torpor depth and duration vary across the estrus cycle in mice, whereby the torpor response is greatest during the diestrus phase in which circulating estradiol is at its peak. Exogenous estradiol lengthens torpor bouts in female mice, but not in males. Knockdown of ERα within the POA blunts torpor in female mice, suggesting that estradiol acting via ERα modulates the activity of hypothalamic neurons that generate torpor. We speculate that this cyclical oscillation in torpor propensity which is lowest in the estrous phase may be an adaptive change that preserves reproduction during periods of moderate environmental stress.

**Supplementary Information:**

The online version contains supplementary material available at 10.1038/s41598-026-41051-y.

## Introduction

Maintenance of core body temperature is an energetically demanding physiological process for warm blooded animals (endotherms) that accounts for a large proportion of daily energy expenditure, particularly in small animals with a larger surface area relative to their volume. For example, mice use around half of their total energy expenditure when maintaining their core body temperature at a typical room temperature of 20–22 °C^1^. Where adverse environmental conditions lead to energy deficit, the metabolic demand of maintaining temperature homeostasis can present a major challenge to survival. A strategy employed frequently across animal species, including mammals, is a controlled and reversible suppression of metabolic activity known as torpor^2,3^. During torpor, metabolism may decrease to as little as 2% of basal rate, and internal body temperature is typically defended at a much lower set point, in some species dropping to near ambient temperatures, even in freezing conditions^4,5^.

There is considerable interest in artificially inducing torpor-like states for potential human applications^6–10^. With this in mind, a search for the neural mechanisms responsible for triggering torpor entry has converged on the preoptic area of the hypothalamus (POA) as a key locus for driving this phenomenon in mice^11–17^. Alongside identification of the mechanisms of fasting-induced torpor in mice, it has been shown that neurons in the POA may also drive torpor-like states in rats, which do not naturally enter torpor on fasting^14,16,18–20^; another group has achieved a synthetic torpor-like state in rhesus macaques, demonstrating that synthetic torpor may be achievable in primates^21^.

An interesting observation is that many torpor studies primarily or exclusively use female mice. It has been suggested that female mice more readily enter torpor and are thus preferred as experimental subjects for their consistency^22^. Despite this observation, the effects of sex or indeed circulating sex hormones on torpor response has not been closely examined. Estrogens, principally estradiol, modulate thermoregulation by tuning BAT thermogenesis and vasomotor activity^23,23,24^. Estrogens cross the blood brain barrier and can exert their thermoregulatory effects via central mechanisms^25,26^. Indeed, estrogen receptors (ERs) are expressed throughout the central thermoregulatory pathways and are particularly enriched within the preoptic area (POA) of the hypothalamus^27,28^. Neurons in the POA that induce torpor have a distinct transcriptomic signature, including expression of *Qrfp, Ptger3, Lepr, Opn5*, and *Tacr3* mRNA, which has led to them being termed the QPLOT neurons. These QPLOT neurons co-express a host of receptors including ERα, positioning them to integrate hormonal, metabolic, thermal, and immune inputs that may influence whether an animal enters torpor^29^. Downstream of the POA, ERα is expressed within other hypothalamic regions with roles in thermoregulation and energy balance such as the ventromedial and arcuate nuclei^23,24,30,31^, and is moderately expressed in the dorsomedial hypothalamic nucleus^27^.

The expression of ER in circuits that control both energy balance and thermoregulation, as well as their abundance in the POA, makes them an interesting population for further study. One such recent study demonstrated that ERα^+^ POA neurons are active during fasting-induced torpor and are necessary and sufficient for torpor induction^32^. What remains unclear, however, is whether estradiol signalling via ERα in these neurons influences torpor induction and maintenance, or whether the estrogen sensitivity in these neurons is unrelated to their role in torpor.

This study aimed to explore the sex differences in mouse torpor and to determine whether estradiol modulates entry into or maintenance of torpor. We hypothesised that estradiol levels and ERα-signalling in the POA potentiates torpor. To test this, we fasted female mice to induce torpor at each of the three phases of the estrus cycle where estradiol levels are naturally oscillating (and compared to males under equivalent conditions); additionally, we examined the effect of exogenous estradiol administration on torpor, and conversely the effect of POA ERα knockdown on torpor.

## Methods

### Ethical statement

Experiments were conducted according to the guidelines, and with approval, of the University of Bristol Welfare and Ethical review board and in accordance with the Animals (Scientific Procedures) Act 1986, under a UK Home Office Project Licence. This study is reported in accordance with current ARRIVE guidelines for the reporting of animal experiments.

### Mice

For all experiments, C57/BL6j mice were used. Adult (60–100 day old) mice were group housed unless otherwise stated, with ad libitum access to water and standard rodent chow. Mice were housed in a 12h:12h light:dark cycle (lights on at 10:00 am, ZT + 0 h) at a standard temperature of 21 °C ± 1 °C. At the point of entry to experiment, the mean body mass was 21.7 ± 0.5 g for female mice, and 25.9 ± 0.7 g for male mice.

### Estrous cycle staging

The mouse estrous cycle was determined by vaginal cytology as described previously^33^. Briefly, daily vaginal lavage was performed with ~ 20 µL sterile saline for a period of up to 14 days, flushing the canal several times to obtain a sufficient sample, then transferring 2–3 drops onto a glass slide. Samples were allowed to air dry and then stained with 0.1% crystal violet for 5 min before washing with ddH_2_O and cover slipping. Specimens were assessed by brightfield microscopy with the relative presence of leukocytes, cornified or nucleated epithelial cells being used to determine the stage of estrous on the day of sample collection. Examples of cytology present during diestrus, estrus and proestrus are shown in Supplementary Fig. 1.

### Torpor induction and thermography

For recording of torpor induction, mice were transferred into custom built Perspex chambers, each containing inserts that divided the space into quadrants and allowed simultaneous recording of four mice per chamber (as per^11^). On the day of recording, mice were placed into the chamber at ZT + 7 h, initiating the 24 h fast. Where mice were recorded multiple times over the estrus cycle, the order was pseudorandomised by initiating the first torpor bout at different phases of the estrus cycle between mice. When multiple recordings occurred, a minimum of four days recovery was allowed between fasts. Surface body temperature was recorded using a FLIR C2 thermal camera (Teledyne FLIR LLC) positioned above the recording chamber. Temperature data was obtained from captured footage using ResearchIR software (FLIR), using the region of interest (ROI) and maximum temperature functions.

### Quantification of torpor bouts

Further analysis of surface temperature data was carried out using MATLAB R2021a software (The MathWorks Inc., https://www.mathworks.com). Briefly, data outputs from ResearchIR were imported into MATLAB where timestamps and maximum temperature within each ROI per frame of video were imported. We defined a bout of torpor as any period in which surface temperature dropped below 29 °C for ≥ 30 min. Using these criteria, we calculated the duration of time spent in torpor during the 24 h period, and the time to onset of the first torpor bout from the start of the recording period. Euthermic baseline temperatures were calculated by the mean surface temperature during the first three hours of recording and subsequently, Δ temperature from euthermic baseline to the nadir reached during torpor was calculated.

### Estradiol treatments

A stock solution of 17-β-estradiol (cat# 8875, Sigma-Aldrich) was prepared by first dissolving 1 mg/mL in absolute ethanol, which was then added to 50 mL of chen oil; the ethanol was evaporated within a vacuum centrifuge for ~ 60 min giving a final stock solution of 20 µg/mL. Male and female mice were treated with 2 µg / 20 g body mass s.c. bolus (or chen oil vehicle), administered at 10:00 am (ZT + 0 h) to ‘mimic’ the diestrus peak of estradiol described in C57/BL6 mice recently^34^. Female mice were treated on days they were in estrus, determined by vaginal cytology as described above. Each animal received both estradiol and vehicle in randomised order prior to fasting with a minimum 4 days between treatments/fasts.

### Viral constructs

Lentiviral vectors were obtained from Santa-Cruz Biotechnology (Lenti-shERα; cat# sc-29306-V) prepared at a titre of 1.0 × 10^6^ Infectious Units per millilitre (IFUs). This vector contained four *Esr1-*specific constructs encoding 19–25 nt short hairpin RNAs designed to knock down the expression of ERα in mouse tissues^35^.

The AAV5-GFP (pAAV.CMV.PI.EGFP.WPRE.bGH) was obtained from Addgene (RRID:Addgene_105530, a gift from James M. Wilson), at a titre of 7.0 × 10^12^ viral genomes per millilitre (vg/mL). AAV5-GFP delivers the EGFP gene under the control of the constitutively expressed CMV promotor and was used to assess the placement of stereotaxic injections.

### Stereotaxic viral injections

Mice were anaesthetized with ketamine (Ketavet; 70 mg/kg, i.p.) and medetomidine (Domitor; 0.5 mg/kg, i.p.) and maintained with additional injections if needed. Following preparation of the surgical site, mice were placed in a stereotaxic frame (Model 963 Small Animal Stereotaxic Instrument, David Kopf Instruments) and their body temperature maintained using a heat pad (Harvard Apparatus). Glass pipettes were filled with mineral oil and affixed onto a robotic microinjector (Nano-W wireless capillary microinjector, Neurostar). For ERα knockdown injections, pipettes were backfilled with Lenti-shERα, mixed 3:1 with AAV5-GFP to delineate the injection site (n = 7). For control injections, pipettes were backfilled with AAV5-GFP diluted 1:3 in sterile 0.9% saline per mouse (n = 8). The resulting injected viral titres were 7.5 × 10^5^ IFUs for Lenti-shERα and 1.75 × 10^12^ vg/mL for AAV5-GFP.

Burr holes were made at AP + 0.4 mm, ML ± 0.5 mm relative to Bregma. Bilateral injections were then made at DV -5.1 mm and -5.0 mm relative to the surface of the brain, targeting the medial POA. Virus was delivered at 62.5 nL/min to a final volume of 125 nL per injection site and therefore 250 nL per hemisphere. Within each hemisphere, 2 min were allowed before delivery of the second injection; following the second injection, 5 min were allowed before retraction of the pipette. Atipamezole (1 mg/kg, i.p) was used to reverse anaesthesia and carprofen (5 mg/kg, s.c.) was given for pain management up to 3 days post-surgery. Mice were individually housed for five days following surgery and were allowed to recover for a least two weeks following surgery before commencing experiments, torpor was assessed between two- and four-weeks post-surgery.

### Tissue collection and immunohistochemistry

Mice were killed with a terminal dose of pentobarbital (Euthetal; 175 mg/kg, i.p.), followed by transcardial perfusion of heparinised saline (~ 15 mL, 50 units/mL heparin in 0.9% NaCl) and 15–20 mL neutral buffered formalin (VWR Chemicals). Brains were removed and stored in 10% formalin overnight, before being transferred to a 30% sucrose solution with 0.02% sodium azide and stored at 4 °C. Coronal brain slices (30 µm thickness) containing the POA were collected using a freezing microtome and mounted onto Superfrost Plus slides (Epredia). Slices were washed twice for 2 min in PBS-T (PBS + 0.1% Triton), then blocked with 0.25% bovine serum albumin and 10% normal donkey serum in PBS-T for 1 h at RT. Slices were then incubated with a primary rabbit anti-ERα antibody (1:1000; Merck Millipore, cat# 06–935, RRID: AB_310305) overnight at 4 °C. Slices were then washed three times for 5 min in PBS-T, then incubated with secondary donkey anti-rabbit 568 for two hours at RT. Slices were washed, counterstained with DAPI (1:10,000) for 5 min, then cover slipped using Fluoromount G hard set mounting medium.

### Microscopy and image analysis

Images of sections throughout the POA were collected using an Olympus VS200 slide scanner microscope. Image files were registered against the Allen Mouse Brain Common Coordinate Framework^36^ (https://atlas.brain-map.org/) using the Aligning Big Brains & Atlases (ABBA; https://biop.github.io/ijp-imagetoatlas/) plugin for Fiji^37^. Registered images were further analysed in QuPath^38^ (https://qupath.github.io/) using the cell detection function to quantify ERα^+^ cells. Cell count .csv files were exported and collated in RStudio (R version 4.2.1) and pre-processed to determine the total ERα^+^ cells in POA nuclei per mouse before statistical analysis.

### Statistics

Statistical analysis and data visualisation was performed in RStudio and GraphPad Prism 10 (GraphPad Software). Power calculations for assessing group sizes for the ERα knockdown study were performed in G*Power^39^. Prior to any statistical comparisons, normality was assessed by Shapiro–Wilk test. The effect of estrous cycle stage on female mouse torpor was compared using one-way repeated measures ANOVA with Tukey’s multiple comparisons tests. Within-animal comparisons of vehicle and estradiol treatment effects on torpor was conducted by Two-Way ANOVA and Šidák’s multiple comparisons tests. Comparisons between torpor in male and female mice and virally treated control vs knockdown mice were carried out by unpaired two-tailed t tests. Comparison of male and female mice factoring body mass as a covariate was performed by ANCOVA. Results are presented as either mean ± SD or median and quartiles (as appropriate). *P* values ≤ 0.05 were considered statistically significant.

## Results

### Depth, duration and latency of torpor varies through the mouse estrus cycle

**Fig. 1:**
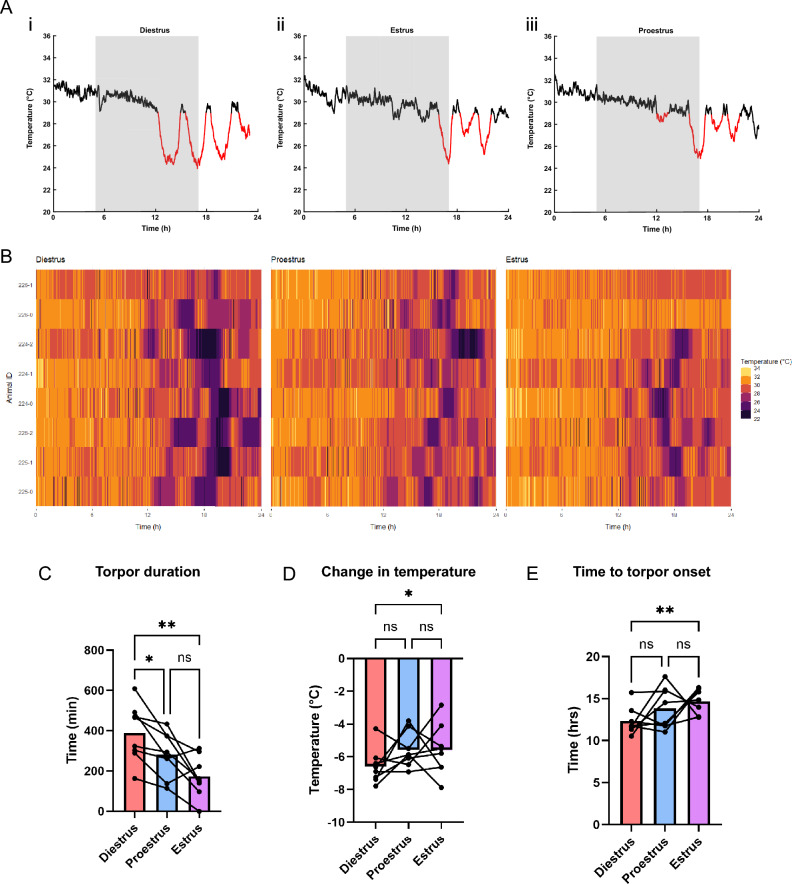
Torpor depth and duration vary over the course of the female mouse estrous cycle. (**A**) Representative surface temperature plots from one fasted mouse during diestrus, proestrus and estrus (torpor bouts highlighted in red defined as surface temperature < 29 °C for > 30 min, grey boxes represent lights off). (**B**) Heatmaps representing a gestalt overview of temperature over 24 h following fasting from 8 mice in diestrus, proestrus and estrus. (**C**) Total duration in hours spent in torpor during a 24-h fast over the estrous cycle. (**D**) The surface temperature change from euthermic baseline to torpor nadir in each phase of the estrous cycle. (**E**) The time to onset of the first bout of torpor during a 24-h fast in each phase of the estrous cycle. Histograms show means. *p ≤ 0.05, **p ≤ 0.01.

To assess the effect of the estrous cycle on torpor, induced by a 24-h period of fasting, we recorded surface temperature in female mice (n = 8) across the diestrus, proestrus and estrus phases (Fig. 1A, B). Mice in diestrus exhibited deeper torpor bouts sustained for longer, compared to the same mice in proestrus and estrus (Fig. 1A, B). We found the duration of time spent in torpor was significantly influenced by estrous cycle stage (Fig. 1C, RM one-way ANOVA, F_(2, 23)_ = 12.75, p = 0.0021) where mice spend longer in torpor during diestrus (389.3 ± 143.3 min) than in proestrus (282.1 ± 113.4 min, p < 0.01) or estrus (172.8 ± 103.2 min, p < 0.05). Similarly, estrous cycle stage also significantly influenced the magnitude of temperature fall from euthermic baseline during bouts of torpor (Fig. 1D, RM one-way ANOVA, F_(2, 23) =_ 2.362, p = 0.043) where there was a greater reduction temperature in diestrus (-6.59 ± 1.08 °C) vs. estrus (-5.6 ± 1.57 °C, p < 0.05, Tukey’s post hoc). The stage of the cycle also impacted the time to the onset of the first torpor bout following the removal of food (Fig. 1E, RM one-way ANOVA) where onset of torpor was significantly earlier in diestrus (12.32 ± 1.62 h after food removal) compared with estrus (14.67 ± 1.51 h, p < 0.01, Tukey’s post hoc).

### Female mice display longer and deeper bouts of torpor than males, mediated by body weight

**Fig. 2:**
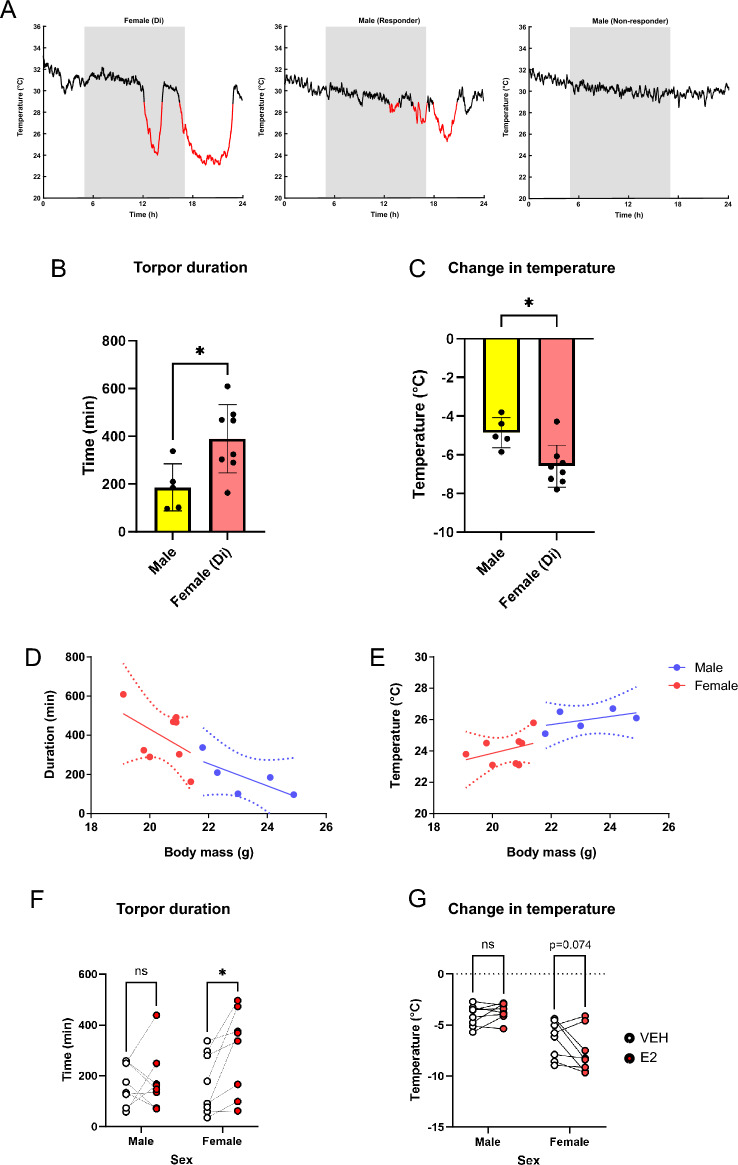
Female mice display deeper and longer torpor bouts that are potentiated by exogenous estradiol administration. (**A**) Representative surface temperature plots from from female, responder male, and non-responder male mice, (torpor bouts highlighted in red defined as surface temperature < 29 °C for at > 30 min, grey boxes represent lights off). (**B**) Total duration of time spent in torpor in male vs. female mice. (**C**) Nadir temperature change from euthermic baseline in male vs. female mice during torpor plotted against body mass. (**D**-**E**) Body weight against torpor duration (**D**) and nadir surface temperature (**E**), showing that differences in torpor characteristics are driven by body temperature. (**F** – **G**) the effect of exogenous estradiol on duration and change in temperature from euthermic baseline in male vs. female mice (open circles control, filled circles – with estrogen). Histograms show mean ± SD. Asterisks denote significant differences by Student’s t-test (**B**, **C**) or Šidák’s multiple comparisons test (**F**, **G**). *p ≤ 0.05, **p ≤ 0.01.

We assessed sex differences in torpor by comparing female mice in diestrus to male mice over the course of a 24 h fast. Interestingly, just five out of eight male mice entered torpor compared to all the female mice indicating that male mice may have a lower probability of entering torpor when challenged with a single fasting period (Fig. 2A). Where torpor did occur, female mice sustained torpor for a longer duration than males (Fig. 2B, 389.3 ± 143.3 vs. 186.1 ± 98.2 min; t = 2.77, p = 0.018, Unpaired t-test), and reached cooler temperatures from euthermic baseline (Fig. 2C, -6.59± 1.08 °C vs. -4.8 ± 0.78 °C; t = 3.10, p = 0.01, Unpaired t-test). At the time of experiment, the age matched male mice had greater body mass than females (23.2 ± 1.28 g vs. 20.5 ± 0.77 g, t = 2.61, p < 0.001, Unpaired t-test). Accordingly, factoring body mass as a covariate, there was a significant effect of body mass, but not sex, on both torpor duration (body mass: F_(1, 10)_ = 13.4, p = 0.0044; sex: F_(1, 10)_ = 0.018, p = 0.89, ANCOVA) and nadir temperature (body mass: F_(1, 10)_ = 16.9, p = 0.0021; sex: F_(1, 10)_ = 1.44, p = 0.26, ANCOVA). This was confirmed by comparison of body mass adjusted means for duration (males: 301.3 ± 78.4 min vs. females: 317.3 ± 54.9 min, t = 0.137, p = 0.89, unpaired t-test) and nadir temperature (males: 25.4 ± 0.57 °C vs. females: 24.4 ± 0.40 °C, t = 1.20, p = 0.26, unpaired t-test).

### Exogenous estradiol treatment lengthens torpor bouts in female, but not male mice

We next tested the effect of exogenous estradiol on fasting-induced torpor in male mice and in female mice in estrus phase (where estradiol levels are lowest^40^).

Estradiol prolonged torpor duration in response to fasting (p = 0.018), with no sex by treatment interaction (2-way repeated measures ANOVA, p = 0.084). However, post-hoc comparisons showed estradiol significantly prolonged torpor in females (297.0 ± 167.0 min estradiol vs. 169.4 ± 120.4 min vehicle, Fig. 2D Holm–Šidák corrected p = 0.021), but not in males (180.2 ± 118.2 min estradiol vs. 166.8 ± 82.2 min vehicle, Holm–Šidák corrected p = 0.94, Fig. 2F). Estradiol treatment differentially affected torpor depth in males and females. Two-way RM ANOVA revealed no overall treatment effect (2-way repeated measures ANOVA, p = 0.31), however there was a significant Sex × Treatment interaction (Holm–Šidák corrected p = 0.047), indicating that the drug effect differed between males and females. Post-hoc comparisons showed no effect in males (p = 0.69) and a trend toward an increasing the depth of torpor in females (Holm–Šidák corrected p = 0.075, Fig. 2G). Estradiol treatment had no effect on the time to torpor onset in either males or females (2-way repeated measures ANOVA, p = 0.21 for treatment), with no treatment x sex interaction (p = 0.84) and no significant differences when analysed by sex (Supplementary Fig. 2).

### Knockdown of ERα in the POA blunts torpor response following a fast

**Fig. 3:**
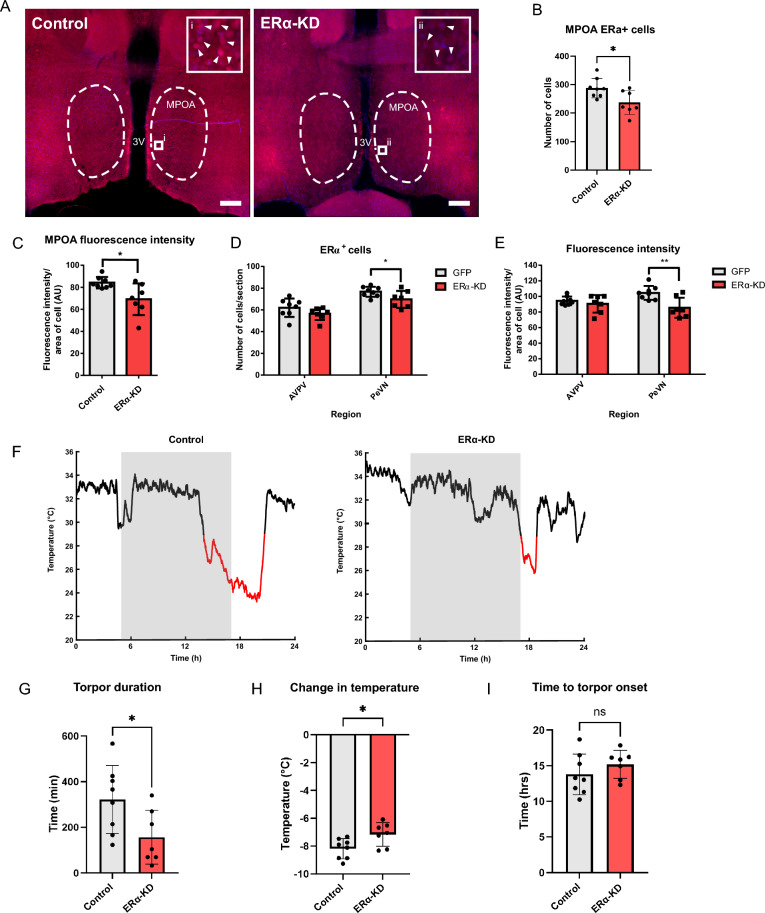
Knockdown of ERα in the POA blunts torpor response following a 24 h fast. (**A**) Representative 20 × photomicrographs taken in the POA of control (left) and sh-ERα injected mice (right) showing ERα (red), with a DAPI counterstain (blue). Dashed lines delineate the medial preoptic area. Scale bar = 100 µm. (**B**) ERα-positive cell counts within the MPOA of control vs. knockdown mice. (**C**) The mean fluorescence intensity per cell area within the MPOA of control vs. knockdown mice. (**D**) ERα-positive cell counts within the AVPV and PeN of control vs. knockdown mice. (**E**) The mean fluorescence intensity per cell area within the AVPV and PeN of control vs. knockdown mice. (**F**) Representative temperature plots from surface temperature thermography recordings in female control and knockdown mice (torpor bouts highlighted in red defined as surface temperature < 29 °C for at > 30 min, grey boxes represent lights off). (**G**) Total duration of time spent in torpor in control vs. knockdown mice. (**H**) Nadir temperature change from euthermic baseline in control vs. knockdown mice during torpor. (**I**) The time to onset of torpor following a 24-h fast. Histograms show mean ± SD. *p ≤ 0.05, **p ≤ 0.01.

Given that activation of estradiol-sensitive neurons in the POA induces a torpor-like state^32^, and our demonstration that exogenous estradiol prolongs torpor in female mice, we hypothesised that the POA is the site at which estradiol modulates torpor. Lentivirus containing short-hairpin RNA to knock down ERα (sh-ERα) was delivered into the POA of female mice and compared with control female mice where only GFP was delivered by AAV. The spread and density of GFP^+^ cells indicated on-target delivery of virus to the medial preoptic area, with limited spread of virus to adjacent areas within the mediobasal hypothalamus, primarily the periventricular nuclei (Supplementary Fig. 3). The number of ERα^+^ cells in the POA of sh-ERα mice was reduced compared to GFP control mice (Fig. 3A, B; 237.1 ± 42.0 cells vs 287.6 ± 34.2 cells, p = 0.023, t = 2.57, Unpaired t-test), and similarly the mean fluorescence intensity of positive cells was reduced in sh-ERα mice compared to controls (84.14 ± 5.14 AU vs. 69.13 ± 14.38 AU; p = 0.016, t = 2.77). Additionally, we assessed knockdown in adjacent areas containing ERα positive neurons that have previously been associated with torpor, namely the anteroventral periventricular nucleus (AVPV) and periventricular nucleus (PeN). Both the number of ERα^+^ cells (76.8 ± 1.6 cells vs. 69.7 ± 2.9 cells; p = 0.049, t = 2.17; Fig. 3D) and ERα associated fluorescence intensity (104.5 ± 3.0 AU vs. 85.5 ± 4.9 AU; p = 0.005, t = 3.38; Fig. 3E) was significantly reduced in the PeN, but not the AVPV. Surface temperature thermography profiles during fasting in ERα knockdown mice were distinct from control mice (Fig. 3F). ERα knockdown mice had shorter torpor bouts (Fig. 3G; p = 0.035, t = 2.35, Unpaired t-test), as well as shallower bouts (Fig. 3H; -7.16 ± 0.85 °C vs. -8.19 ± 0.72 °C; p = 0.024, t = 2.55, unpaired t-test). In contrast, ERα knockdown did not change the time to onset of torpor following fasting (Fig. 3I).

## Discussion

This study examined the causal role of estrogen signalling in controlling torpor in the mouse. We observed that female mice exhibit deeper and longer bouts of torpor during the phase of their estrous cycles where estradiol is highest. We confirmed that estradiol signalling mediates this association: exogenous estradiol lengthened torpor bouts in female mice (but not males). Finally, we show that knocking down ERα reduced torpor length and depth in female mice, and we speculate that estradiol exerts this effect preferentially via ERα-positive neurons in the POA. Together, these results build on the growing body of work implicating the POA as a key site for torpor induction, and indicate that in female mice, activity of POA torpor-inducing neurons is enhanced by the action of estradiol on ERα.

We found that while males exhibit shorter and shallower bouts of torpor, this difference can be entirely explained by body mass. We did observe that exogenous estradiol administration prolonged the duration of torpor in female but not in male mice, suggesting that estrogen signalling differentially modulates torpor in males and females. Such a sex difference is plausible given the POA is known to be sexually dimorphic^41^, although it is also possible that any effect of exogenous estradiol administration was masked by dominant body mass effects in males.

ERα may influence activity and excitability of neurons through both genomic and second messenger mediated pathways. To act via the latter, ERα may be trafficked to the plasma membrane via post-translational palmitoylation and subsequent association with the scaffold protein calveolin-1^42,43^, where it forms functional dimers that associate with G-protein complexes^44^. Membrane associated ERs facilitate estradiol’s rapid modulation of cellular processes by activating secondary pathways including metabotropic glutamate receptors^45^. Functionally, this has mostly been studied in the context of cognition, memory and motivation, however, there is evidence that estradiol acting via metabotropic glutamate receptors in the medial POA decreases food intake^46^. ERs also function as intracellular receptors capable of modulating neural activity and excitability by stimulating calcium release from the endoplasmic reticulum, or by modulating ion channel activity^47^. Whether this leads to up or down-regulation of excitability is context dependent. For example, again with respect to energy balance in the hypothalamus, estradiol disinhibits pro-opiomelanocortin neurons by attenuating GABA_B_-mediated activation of inwardly-rectifying potassium channels, while the opposite is true of agouti-related peptide neurons where GABAergic inhibition is enhanced^48^ Hence, estradiol is capable of acting in the hypothalamus to modulate neural activity and change behaviour via multiple cellular pathways.

Our results support the hypothesis that, in the case of POA torpor-inducing neurons, the effect of ERα activation is to excite the neurons. Studies of neurons in the POA consistently report that their activation drives torpor^13,14,32,49^, and POA neural activity is elevated during torpor when examined by cFos expression or in vivo recording of calcium events by fibre photometry^11–14^. It follows then that estradiol increasing excitability of neurons in the POA would promote torpor.

It is not known whether initiation and maintenance of torpor are distinct processes, or whether a single population of neurons trigger torpor, which is then maintained for as long as the neurons are active. An intervention that affects time to torpor onset (or likelihood of torpor) without affecting torpor duration (or vice versa) would support the hypothesis that initiation and maintenance or governed by independent systems. We found that time to torpor onset was shorter when circulating estrogen is highest during diestrus than when it is lowest during estrus. At this phase in the cycle, torpor duration was longer and depth was greater, hence onset and maintenance covary. In contrast, manipulation of estrogen signaling in females, either through exogenous administration of estradiol or by estrogen receptor knockdown that targets the POA, did increase torpor duration without affecting time to onset. How might we explain these divergent results whereby time to torpor onset and duration of torpor covary across the natural estrous cycle in females, but manipulation of estrogen signalling only increases duration? In the case of estradiol treatment, we might have delivered the wrong dose at the wrong time to observe an effect on time to torpor onset. In the case of estradiol receptor knock down, perhaps we had insufficient knockdown of ERα. We achieved a knockdown of 17.8% based on fluorescence intensity. This degree of knockdown was enough to perturb torpor duration and nadir temperature, but a more robust knockdown may be needed to affect the time of onset. Alternatively, estrogen might affect the time to torpor onset through actions outside of the POA. Regardless, these experiments were not designed to determine the relationship between torpor onset and maintenance, and further studies are needed to further elucidate this relationship. Because in our 24-h fasting model we found that all females entered torpor (regardless of cycle phase), our data cannot inform on whether estradiol signalling modulates the probability of torpor.

One question raised by our findings across the sexes is why estradiol enhances torpor in female mice, but not males. The POA displays sexual dimorphism: several nuclei differ in size, neurotransmitter expression, and cell number^50^. Furthermore, ERα expression is greater in the POA of female mice compared to males^32^. Female mice may have adapted to become more energetically thrifty, making ‘savings’ in diestrus when they are not fertile. This might allow for greater energy expenditure with shorter and shallower torpor bouts typically seen during proestrus and estrus, which is when mating occurs. Hence, female mice preferentially allocate energy expenditure to periods when mating occurs and prioritise energy saving when mating does not occur. An interesting consideration is whether stimuli such as the presence of male scent-marked bedding across the estrous cycle has any effect on torpor, which may allow us to tease apart the hierarchy of priorities between mating and energy savings. Alternatively (or additionally), the low metabolic activity and body temperature of torpor might be hazardous during early pregnancy, in which case torpor might be inhibited during periods of the cycle in which fertilisation and implantation occur. In this interpretation, torpor is not so much promoted during diestrus as inhibited at other times.

Energy expenditure and food intake are additional factors that fluctuate over the course of the estrous cycle. Energy expenditure is lower in diestrus compared to estrus in both rats and mice and diestrus is associated with lower heat production over 24 h in mice^51^. Food intake, similarly, may vary over the course of the estrous cycle by 20% and is highest during estrus^52^. It is possible that mice experience a period of relative energy deficit during diestrus compared to other phases of the cycle. This might make torpor more advantageous at this timepoint. Hence, the POA torpor-inducing neurons might have evolved estrogen-sensitivity to align torpor with periods of the estrus cycle in which relative energy deficit occurs.

### Limitations

We mapped the injection site of the viral vector using expression of EGFP fluorescence using a co-injected AAV. This was necessary as the promoter for the shRNA does not allow expression of fluorophore transgene. It should be noted that this AAV will have had a different cellular tropism, promoter and diffusion characteristic to the lentiviral vector and so cannot be used to definitively infer the extent and identity of cells transduced by the lentivirus^53^. This was mitigated to a degree by mapping the extent of knock down of the ERα protein using immunocytochemistry. Furthermore, lentivirus titre was 1.0 × 10^6^ Infectious Units per millilitre vs 7.0 × 1012 for the AAV, hence the lentivirus was ~ 1 million times lower titre and is therefore unlikely to have spread further than the EGFP vector. We also only injected the lentiviral vector to the intervention group meaning we did not control for any possible effects of the lenti-vector itself mediated directly on neurons or by a localised immune reaction – however these vectors have been used extensively to transduce central neurons because they are well tolerated and evade the immune system.

Another consideration is that our focus on estradiol does not consider other sex hormones that might have additional roles in regulating torpor. Progesterone levels are lower throughout most of the mouse estrous cycle aside from a brief peak in late proestrus^40^, likely generated by the corpus luteum following ovulation^54^. While progesterone released during the luteal phase, and taken exogenously from oral contraceptives, drives an increase in body temperature in humans (~ 0.5 °C)^55^, its role in mice and other animal models is not as clearly defined. One study in rabbits reported that progesterone drove an increase in body temperature, with a concurrent decrease in firing rate from warm-sensitive POA neurons^56^. Other studies in rats suggest however, that progesterone treatment does not alter sympathetic output to brown adipose tissue^57^, a principal mechanism by which central circuits modulate thermogenesis. Progesterone is released in a relatively confined window of early estrus before being rapidly metabolised^54^ in cycling adult female mice, so may have a brief window of influence on torpor during this phase. The role of progesterone on torpor and indeed, thermoregulation more broadly, is currently poorly understood and is a clear target for studies examining the role of sex hormones in torpor going forward.

## Conclusions

These findings highlight estradiol as a key modulator of torpor, both deepening and prolonging the torpor state through its actions on ERα-positive neurons, including those in the POA. Further work to define how estradiol influences specific neuronal populations in this region will be crucial for understanding how these circuits integrate physiological signals to orchestrate torpor. More broadly, this study demonstrates that estrogen shapes the fasting response in female mice, likely via POA neurons, and that torpor propensity varies across the estrous cycle. Beyond the context of mouse torpor, these results offer important insight into the central mechanisms linking energy balance and reproductive state—an interaction that is also highly relevant to human reproductive physiology.

## Supplementary Information

Supplementary Information.

## Data Availability

The datasets used and analysed in the current study will be made available upon reasonable request to the corresponding author.
