# Supplementary material for: Estrous cycle modulates fasting-induced torpor propensity via hypothalamic estrogen signalling
Source: Sci Rep. 2026 Feb 27;16:11214. doi: 10.1038/s41598-026-41051-y (PMC13047001; doi:10.1038/s41598-026-41051-y)
Supplement: Supplementary file 1 — Supplementary Information. [file 41598_2026_41051_MOESM1_ESM.docx]

**Supplementary Figure 1. Vaginal cytology across the estrous cycle.** Example micrographs showing the vaginal cytology samples collected in each animal during the diestrus, proestrus and estrus phases of the estrous cycle.

**Supplementary Figure 2. Time to torpor onset with estradiol treatment vs vehicle.** There were no significant differences in time to torpor onset and no sex x treatment interaction. Males entered torpor later than females (2-way repeated measures ANOVA, p <0.0001).


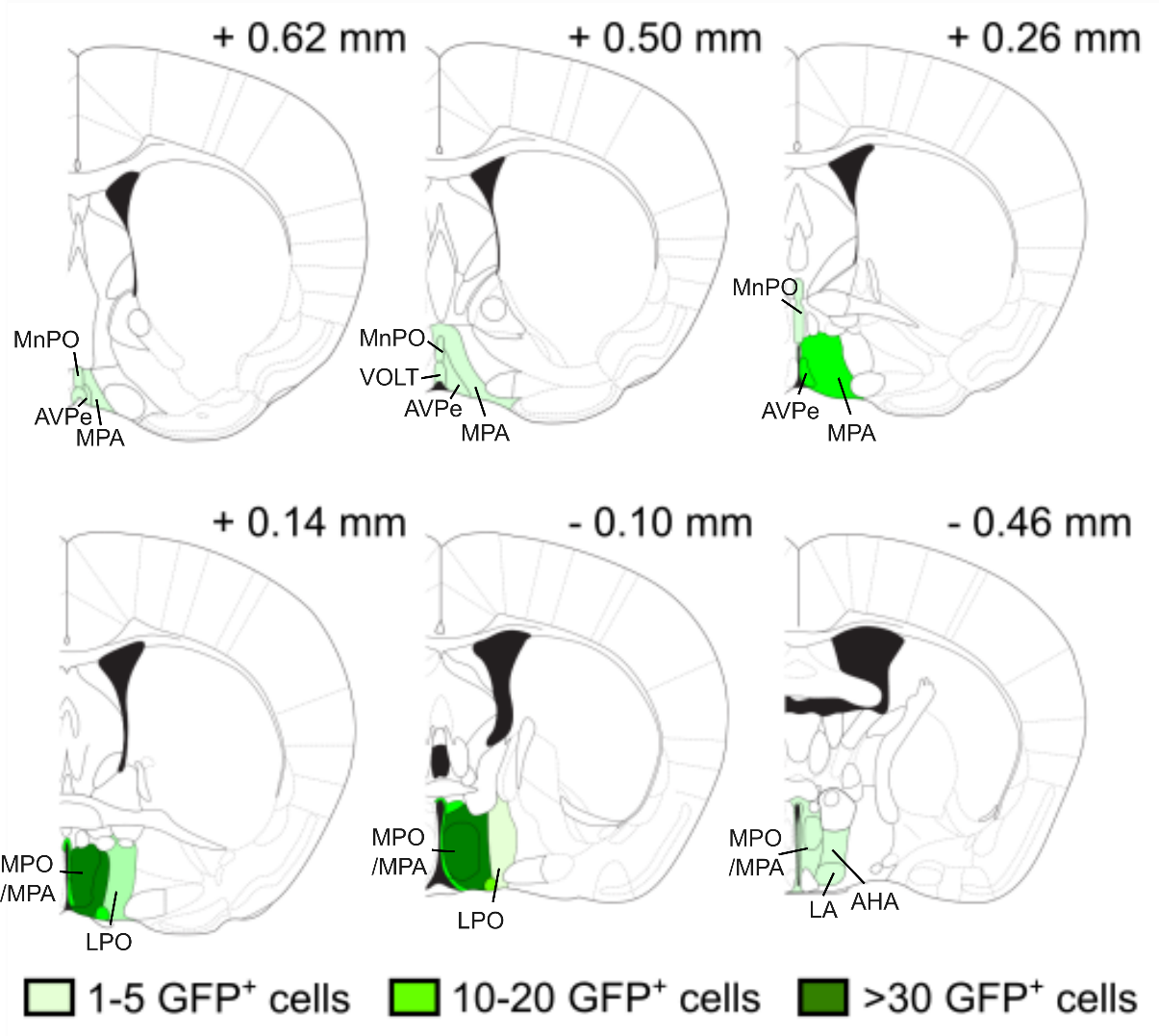


**Supplementary Figure 3. Distribution of GFP-positive cells through the preoptic area.** The mean number of GFP-positive cells detected within nuclei and areas of the preoptic area (per section per mouse, n = 15) is represented semi-qualitatively in light, medium and dark green. Abbreviations: MnPO, median preoptic nucleus; AVPe, anteroventral periventricular nucleus; MPA, medial preoptic area; VOLT, vascular organ of the lamina terminalis; MPO, medial preoptic nucleus; LPO, lateral preoptic nucleus; LA, lateroanterior hypothalamic nucleus; AHA, anterior hypothalamic area anterior part.
